# Local-Entropy Based Approach for X-Ray Image Segmentation and Fracture Detection

**DOI:** 10.3390/e21040338

**Published:** 2019-03-28

**Authors:** Franko Hržić, Ivan Štajduhar, Sebastian Tschauner, Erich Sorantin, Jonatan Lerga

**Affiliations:** 1 Department of Computer Engineering, Faculty of Engineering, University of Rijeka, 51000 Rijeka, Croatia; 2 Division of Pediatric Radiology, Department of Radiology, Medical University of Graz, 8036 Graz, Austria

**Keywords:** bone fracture detection, image segmentation, image classification, local entropy, Shannon entropy

## Abstract

The paper proposes a segmentation and classification technique for fracture detection in X-ray images. This novel rotation-invariant method introduces the concept of local entropy for de-noising and removing tissue from the analysed X-ray images, followed by an improved procedure for image segmentation and the detection of regions of interest. The proposed local Shannon entropy was calculated for each image pixel using a sliding 2D window. An initial image segmentation was performed on the entropy representation of the original image. Next, a graph theory-based technique was implemented for the purpose of removing false bone contours and improving the edge detection of long bones. Finally, the paper introduces a classification and localisation procedure for fracture detection by tracking the difference between the extracted contour and the estimation of an ideal healthy one. The proposed hybrid method excels at detecting small fractures (which are hard to detect visually by a radiologist) in the ulna and radius bones—common injuries in children. Therefore, it is imperative that a radiologist inspecting the X-ray image receives a warning from the computerised X-ray analysis system, in order to prevent false-negative diagnoses. The proposed method was applied to a data-set containing 860 X-ray images of child radius and ulna bones (642 fracture-free images and 218 images containing fractures). The obtained results showed the efficiency and robustness of the proposed approach, in terms of segmentation quality and classification accuracy and precision (up to 91.16% and 86.22%, respectively).

## 1. Introduction

Various image processing techniques are frequently used for enhancing images produced by medical image acquisition systems, such as Digital Radiography (DR), Sonography, Computed Tomography (CT), or Magnetic Resonance Imaging (MRI). Diagnostic accuracy, in terms of ability to detect and classify abnormalities in these images, depends highly on image quality. In the case of low-quality images, numerous image processing techniques utilise information entropy as a tool for image feature extraction [1]. For instance, entropy is of great use in studying and examining the liver and other soft tissue organs [2,3,4].

Modern studies in medical imaging strive to develop efficient computer-aided decision support systems to assist medical experts by flagging suspicious cases requiring closer examination [5]. The goal of these automatic computerised diagnostic systems is to reduce diagnostic time and improve accuracy by replacing the tedious inspection of medical images (traditionally done visually by human experts) with computerised software solutions [6,7]. However, the current computer-aided systems are not able to replace medical staff entirely, but rather assist them by directing their attention to suspicious cases.

Bone fracture detection is a growing research field. Due to the diversity in shapes and types of bones, numerous methods focus only on specific bones. Namely, some fracture detection algorithms are based on inspecting bone orientation, while others search for irregularities in bone texture [8,9,10]. Newer methods involve training deep neural networks which require large quantities of (often unavailable) data for training [11]. Furthermore, the input data for training neural networks must be properly labelled, especially when used for the localisation of the regions of interest. Such labelling is time-consuming and requires expert medical knowledge.

In addition, the automatic computerised analysis of medical imaging can be used in telemedicine as a practical solution for acute shortages of medical practitioners in rural areas. Namely, with the aid of software-based diagnostics of X-ray scans, paramedical staff in remote areas would be able to identify the severity of injuries and respond adequately in a timely fashion [12,13].

Fractures of the ulna and radius bones are common injuries in children [14]. A radiologist can easily overlook smaller fractures of the ulna and radius bones, as children’s bones are continuously growing. For that purpose, a system for fracture detection would make discovering smaller fractures easier, reducing the chances of a false-negative diagnosis in children. To the best of our knowledge, there exists no published work dealing with these types of fractures using local entropy approaches (such as the one developed in this paper).

This paper proposes a hybrid computer-aided X-ray image segmentation and bone fracture detection solution, which consists of the following phases: (1) Directional alignment; (2) entropy-based bone contour extraction and denoising; (3) applying graph theoretic techniques in bone contour correction and fracture detection; and (4) a classification and localisation procedure. The proposed method shows that the entropy-based algorithm renders a new perspective for bone contours analysis, which can be useful in discovering fractures. In particular, the proposed algorithm is focused on discovering demanding fractures which can be easily overlooked, since detecting obvious ones (such as open and shattered-bone fractures) is trivial, even for a non-medical observer.

Although some of the method’s phases utilise the existing techniques (such as principal component analysis (PCA) and graph search methods), the proposed pipeline of methods is new and introduces a novel entropy-based bone segmentation concept. The proposed hybrid approach was shown to outperform other existing segmentation procedures for X-ray images. In addition, the given solution was shown to be efficient in not only detection, but also in the accurate localisation of bone fractures.

The rest of the paper is structured as follows: Section 2 provides details on each stage of the proposed multi-stage method. The results of X-ray image segmentation and bone fracture detection are elaborated in Section 3. Conclusions are found in Section 4.

## 2. Methodology

### 2.1. Image Preprocessing

**Figure 1:**
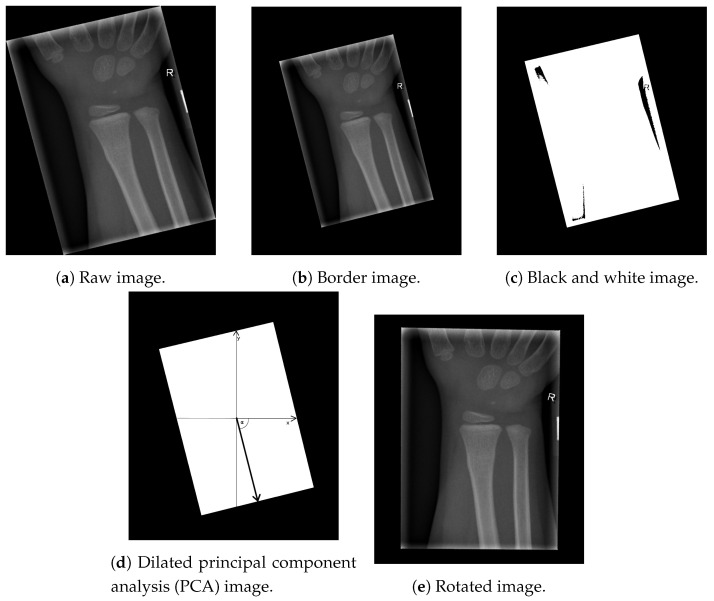
Image alignment.

Image alignment is the first step necessary for an efficient automatic computerised classification procedure of X-ray images, since many patients with fractures of the ulna and radius bones are not capable of aligning their hands properly in an X-ray machine, resulting in angled images (as shown in Figure 1a). Although image alignment is not a problem for visual inspection done by radiologists, it is important in the proposed image segmentation algorithm. Thus, the image alignment stage of the proposed algorithm consists of the following steps:

1. Enlarging the border of the input X-ray image;
2. Transforming the grayscale image into a black and white image;
3. Calculating the vector describing the orientation of the image by utilising the PCA method; and
4. Adjusting the image, so that the vector of the image orientation is aligned with the vertical axis of the background canvas.

In post-processing, clinical X-ray images of children’s extremities should not be electronically cropped beyond the borders of the physical radiation-beam collimation [15], as shown in Figure 1a. The extraction of the resulting angled box coordinates can be improved by enhancing the border between the box and the image frame, which is achieved by inserting a padding of 10 black pixels to the image frame, as shown in Figure 1b. To enhance our region of interest, the image content was transformed to black & white, based on the intensities of the image pixels $P_{intensity}$:

(1) $$P_{intensity}=\left\{\begin{array}{cc} 255 & \;\mathrm{if}\;P_{intensity}>0\\ 0 & \;\mathrm{if}\;P_{intensity}=0 \end{array}\right..$$

The above-mentioned thresholding resulted in the image shown in Figure 1c. It can be seen that the resulting image of this phase contains several artefacts (namely, black regions inside the white box). These artefacts are removed using a morphological closing operation, with a kernel shaped by an all-ones matrix (with dimensions 10×10) [16]. This is a necessary pre-processing step, preceding the box vector calculation, due to its sensitivity to unwanted artefacts.

The next step consists of calculating the angle defining the direction of the box, which represents the region of interest. Utilising traditional methods (such as a Canny filter or Sobel operator) to detect the edges of the box results in edges which can be transformed into vectors [17]. Next, it is necessary to define the centre of the box and the angle between the vector and the horizontal axis of the image. However, instead of white pixels being treated as a part of the box, they are treated as data points. In addition, note that every data-set has defining features (such as standard deviation, variance, mean value, and so on). Here, we employ PCA as a statistical procedure which uses an orthogonal transformation of the original space to convert a set of observations of possibly correlated variables (entities, each of which takes on different numerical values) into a set of values of linearly uncorrelated variables, called principal components [18]. The first principal component has the largest possible variance (to preserve as much variability in the data as possible). In our case, the first principal component is the vector having the same direction as the white box, as shown in Figure 1d (the angle between the vector and the horizontal axis is denoted by α). Once the angle of rotation is calculated, the whole image is rotated around its centre by 90°−α.

Next, the missing pixels caused by rotation are interpolated, using Lanczos interpolation over an 8×8 neighbourhood [19]. The described procedure, with some fine tuning, could be used for aligning any X-ray image whose content is inside of the box. Figure 1e displays an example of an aligned image. The described pre-processing ensures the rotation-invariant property of the proposed computer-aided automatic solution for X-ray image segmentation and fracture detection.

### 2.2. Local Entropy-Based Tissue Removal

A key problem in bone segmentation is the differentiation of tissue from actual bones. Therefore, to solve this problem, we introduce a modified method based on the Shannon entropy (also known as information entropy). The Shannon entropy, previously shown to perform well on a similar problem [20], represents an average rate at which information, usually measured in bits, is produced by a stochastic data source:

(2) $$H\left(X\right)=\sum_{i=1}^{n}P\left(x_{i}\right)I\left(x_{i}\right)=-\sum_{i=1}^{n}P\left(x_{i}\right)log_{b}P\left(x_{i}\right).$$

However, this paper introduces a concept of local (short-term) Shannon entropy. Basically, the idea is to calculate the entropy inside of a sliding window (having dimensions n×n). Namely, the window slides through the picture with a stride of 1 pixel, and local entropy is calculated for each centre pixel, based on all other neighbouring pixels inside the window.

In the Equation (2), P(X) is the distribution of pixel intensities inside the window, *b* is the logarithm base (often set to 2 for bits, as also used in the paper), and n×n is the size of the window (based on the extensive experimental research on training data, *n* was set to 9). The local entropy of border and near-border pixels was calculated by mirroring nearby pixels to fill the missing pixel intensities inside the sliding window. It is important to note that this mirroring is applicable for arm X-rays, because there is usually no tissue or any other useful information near image borders.

The resulting local entropy representation of the image can be treated as a measurement of bone presence. Namely, when pixels inside the window have a similar intensity, the entropy is low. On the other hand, when the bone is captured inside the window with the surrounding tissue, the entropy is high, due to pixel intensity diversity. Next, in order to emphasise this difference, we propose multiplying the local entropy by the standard deviation of the pixels inside the window:

(3) $$V\left(X\right)=\sum_{i=1}^{n}P\left(x_{i}\right)I\left(x_{i}\right)*\sigma \left(X\right)=-\sum_{i=1}^{n}P\left(x_{i}\right)log_{b}P\left(x_{i}\right)*\sigma \left(X\right).$$

**Figure 2:**
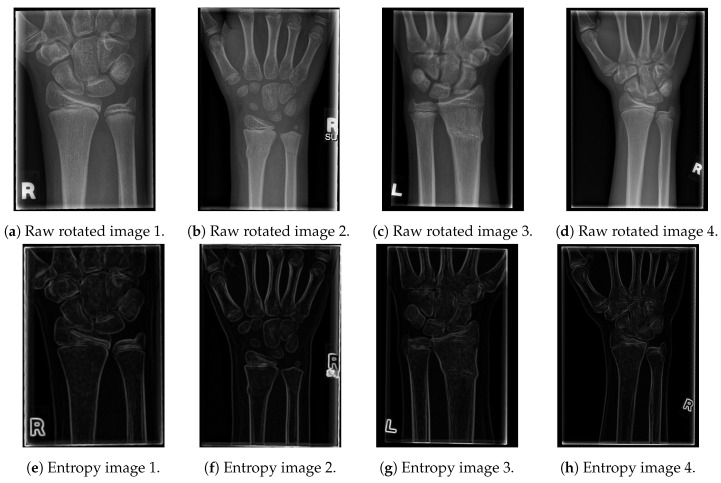
The results of the proposed entropy algorithm on X-ray images.

The resulting images are given in Figure 2.

As shown in the entropy figures, the bone edges remain clearly visible, with only a small amount of tissue and noise left in their vicinity. In order to additionally emphasise the contours of the bones and to obtain a black and white image (with white pixels representing just bone contours), we have tested several edge-detection algorithms and proposed a new one, which outperformed all of them for the tested data-set [21,22]. In particular, the tested algorithms are:

- Canny filter;
- Sobel operator (x and y axis);
- Laplacian edge detector; and
- The proposed algorithm for line-edge detection.

**Figure 3:**
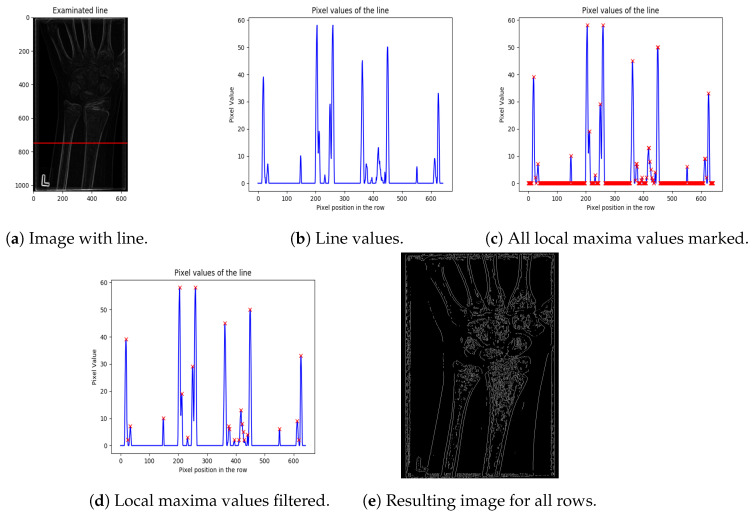
Steps of the line-edge detection algorithm.

The proposed algorithm searches for peaks in pixel intensities for each row of the local entropy image from the previous step of the method (entropy image), where high pixel intensities represent bones. The goal of the proposed algorithm is to preserve high value pixels and to remove low value pixels (representing noise and tissues). In order to do so, the proposed algorithm consists of several steps detailed in sequence (the algorithm is explained for one image row, and the same procedure is repeated for every row of the entropy image):

1. The first step is image normalisation: The image is smoothed by subtracting the mean of all pixel intensities from the original pixel intensity (with negative pixel values obtained by subtraction, being set to 0). This step results in improving the difference between bone and tissue. It also partly removes blurring around the bone, as shown in Figure 3a (the red line in the image denotes a random row chosen for demonstration of this method’s step).
2. The next step examines a distribution of pixel intensities along each row (as shown in Figure 3b, for the red line row denoted in Figure 3a). All large peaks in the graph represent bones in the image. Hence, the next step of the algorithm is to extract the peaks.
3. Peak extraction can be performed by finding the local maxima in the graph values. The local maxima in Figure 3c are denoted by a red “x”. As can be seen, there are a significant number of false positive local maxima detected. Hence, local maxima filtering is required as the next step. Note, also, that a simple thresholding of high value pixel intensities (unlike the low value pixels) is not applicable, since bones are usually represented by several connected pixels in a row, having similar (high) intensities. Namely, thresholding would, in many cases, eliminate both bone contours and false positives.
4. Thus, in order to eliminate false positive peaks, neighbouring pixels having the same or similar intensities, which are marked as the peaks, are combined into one peak. Thus, instead of a “region” of local maxima, only one representative pixel of the region is kept. The representative pixel is placed at the mean coordinate of the pixel region belonging to the representative pixel.
  In addition, zero-valued pixels are removed from bone contours by applying hard thresholding (threshold value is set based on the intensities of pixels larger than 0). Experimental tests using a grid-search algorithm show that a threshold of 20% performs better than mean, median, and quartile thresholds, suppressing most of the noise and preserving all of the peaks which represent bones (as shown in Figure 3d).
5. The final result of the proposed bone extraction procedure for the whole image (all rows of the image) is given in Figure 3e, where the white pixels represent bones.

**Figure 4:**
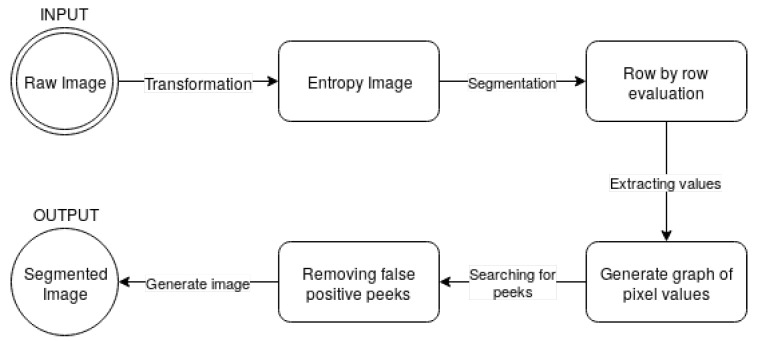
Diagram of the proposed algorithm.

The proposed bone extraction algorithm is also presented in a diagram, shown in Figure 4.

In order to compare the performance of the proposed method to other segmentation and contour-detection algorithms, the noise left after image segmentation (tissue removal) was measured using the global entropy measure (calculated as the sum of image entropy and segmentation entropy).

Namely, the image entropy measures the amount of information left in the image after segmentation (2).

On the other hand, segmentation entropy may be used as a measure for noise artefacts and small-noise fragments left in the image (since a single white pixel or small region of white pixels does not represent a bone). In particular, a bone is represented by a relatively long line of pixels. Hence, measuring artefacts may be reduced to measuring non-bone regions of white pixels done by the segmentation entropy (proposed by J. Hao [23]). The segmentation entropy was defined as the sum of the image entropy E(H), subtracted from the entropy of *n* windows ((E(r)), and divided by the image entropy E(H):

(4) $$E\left(S\right)=\frac{\sum_{i=1}^{n}\left(E\left(r_{i}\right)-E\left(H\right)\right)}{E(H)}.$$

Finally, noise evaluation was obtained by combining the image entropy and the segmentation entropy (scaled by the number of windows):

(5) $$N=H\left(X\right)+E\left(S\right)=\sum_{i=1}^{n}P\left(x_{i}\right)I\left(x_{i}\right)+\frac{\frac{\sum_{i=1}^{n}\left(E\left(r_{i}\right)-E\left(H\right)\right)}{n^{2}}}{E(H)}.$$

All algorithms were first fine-tuned, in order to reach their best performances, on a training set consisting of 100 randomly chosen images from a data-set of 960 images, leaving 860 images as a test set. The hyperparameters for each algorithm, which resulted in its best performance, are as follows:

- Canny filter: The lower and upper borders were set to (1±σ)×μ, where σ=0.66 is the standard deviation of pixels intensities and μ is the mean value of the pixels intensities of the input image;
- Laplacian filter: The dimensions of the standard kernel for the Laplacian filter were set to 3×3; and
- Sobel filters: The dimensions of the kernel were set to 11×11.

The comparison of the proposed method to the Canny filter, Laplacian edge detector, and Sobel operator (both vertical and horizontal) for the test data-set is presented in Table 1. As can be seen, the two methods that stood out among the tested methods are the Canny filter and the proposed method. The Canny filter was negligibly better than the proposed method in artefact detection—by 0.018 bits, in terms of the segmentation entropy. However, the proposed method outperformed the Canny filter by 0.179 bits, in terms of information preserved in the image after segmentation (image entropy). Namely, on the global scale, the Canny filter resulted in a larger number of false positives than the proposed method. When segmentation and image entropies were summed, the global entropy measure was obtained and the proposed method outperformed the Canny filter by 0.161 bits. In other words, the proposed method resulted in an image that preserved the highest number of bone pixels, and removed the highest number of tissue pixels.

### 2.3. Extracting the Region of Interest

**Figure 5:**
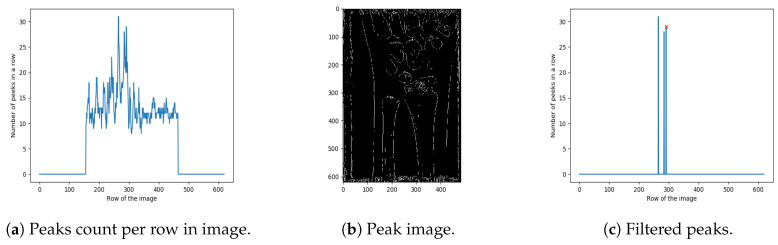
Extracting the region of interest.

The next step in the proposed method is extracting the region of interest (i.e., detecting its boundaries), which in our case contains ulna and radius bones. The bottom boundary is trivial to set (bottom of the image). However, finding the top boundary is based on detecting the correlation between the number of peaks in a row, and the place where ulna and radius bones end. As shown in Figure 5a, there is a large jump in the number of peaks in a row related to the place where radius and ulna bones end (Figure 5b). In order to narrow the search area for the peak with maximum value, the first 25% and the last 25% of the image is cropped out. In general, the ulna and radius bones do not end in the top or the bottom 25% of the real-life X-ray images (if so, the image is considered invalid). Hence, the end of ulna and radius connection is often found close to the image centre [24].

**Figure 6:**
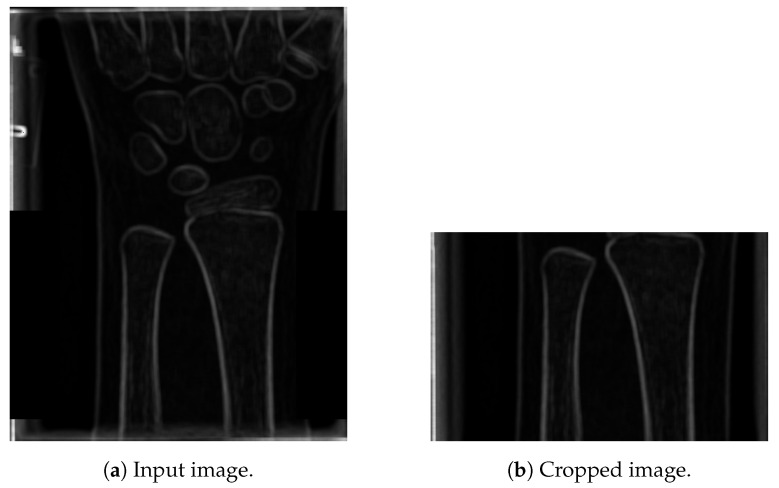
Results of cropping an image by a detected line.

The next step is to extract the highest peaks from the graph, shown in Figure 5a. First, the local maxima regions were found (reduced to a single point/peak detection, as in Step 4). Next, only the peaks higher than 95% of the maximum peak are retained (resulting in just a few such peaks), as shown in Figure 5a. Based on extensive experimental investigation, performed on the 100 images from a training set, it was shown that the far right peak in Figure 5c properly localises the ends of the ulna and radius bones. Namely, the other lines usually represent parts of the wrist. The result of cropping, based on the cropping position detected in Figure 5c, is shown in Figure 6.

Finally, the image width is cropped by finding its left and right boundaries. Width cropping is based on the observation that, usually, peaks representing bones have the highest value. Because two bones are to be detected, four highest values are taken into account. The proposed method for finding the width of the region of interest is based on the average coordinates of four highest values of each row in the previously cropped image. In particular, this procedure can be divided into four steps:

1. Finding the coordinates of four highest peaks of each row in the image;
2. Sorting the coordinates from lowest to highest;
3. Finding the average coordinates values for sorted coordinates;
4. Extracting the lowest and highest coordinates which represent bone boundaries (bone boundaries are expanded by 20% of the bone boundary area, in order to keep some additional area around the bones in the cropped image); and
5. Cropping the image with respect to the set boundary box.

**Figure 7:**
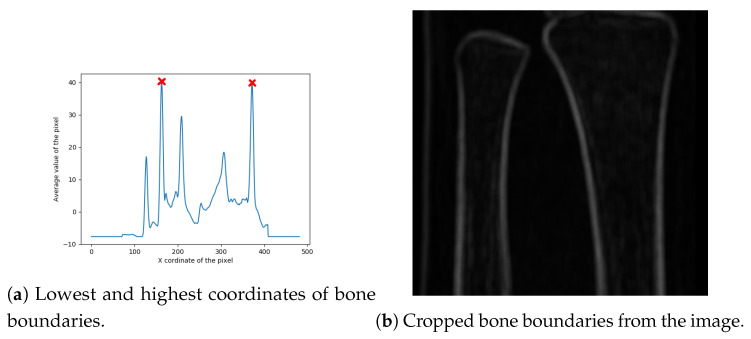
Extracted region of interest.

The red marks in Figure 7a denote the boundary box corners, with the final cropped image presented in Figure 7b.

### 2.4. Bone Extraction

Following the detection of the region of interest, the next step is to create a black and white image of the bone contours. To extract bone contours, we propose a novel method, based on graph theory, utilising several observations specific for long-bone X-rays:

1. The bones are oriented along the vertical axis.
2. Bones start at the very bottom of the cropped image (where, in ideal noise-free images, only four starting points can be found).
3. With regard to the previous observation, for real-life noisy images, false positive lines may be detected, as well as blurred bones, which must be eliminated with an approximation of the actual bone contour.

In order to apply this bone extraction approach to other X-ray images (i.e., not containing long bones), similar regularities and rules should be noticed and implemented. Based on the above-mentioned observations, the proposed graph-based algorithm for bone detection consists of the following steps:

1. Root node selection;
2. Finding the next pixel that belongs to the bone;
3. Building a bone graph; and
4. False bone-pixel elimination.

#### 2.4.1. Root Node Selection

**Figure 8:**
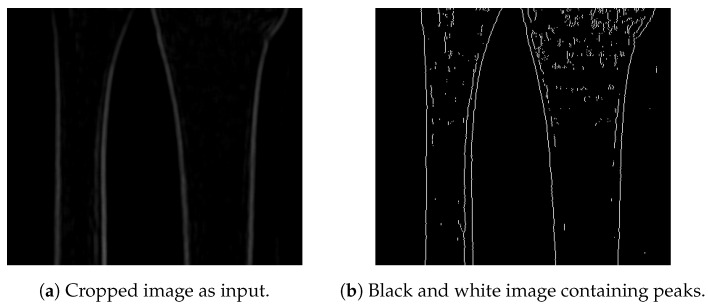
Input image for root detection.

Referring to the presumption that the bones are aligned along the vertical axis, here we introduce a bone detection procedure starting from the bottom of the image (knowing that the next bone pixel is to be found above it). Hence, the root node is to be found at the very bottom of the image. For this purpose, a black and white peak image is generated (shown in Figure 8a for the input image presented in Figure 8b), having white pixels where the intensities of the pixels are the highest.

In real-life scenarios, due to the noise (which is manifested as blurriness), the last line may have more than four white pixels representing the bones. Hence, in this step, all pixels found in the last line are taken as root nodes, and are forwarded to the next step of the algorithm (finding the next pixel that belongs to the bone). In the ideal case, only four pixels are detected as root nodes and, ideally, there are only four lines representing bones. Simulations performed on the training data set have shown that cases with four root notes are rare, and, therefore, image segmentation pre-processing is even more important.

#### 2.4.2. Finding the Next Pixel That Belongs to the Bone

**Figure 9:**
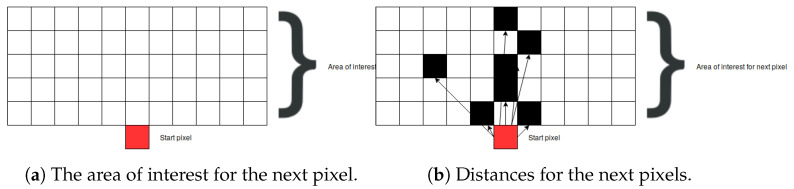
The proposed algorithm for searching for the next bone pixel.

Finding the next pixel from the respective root node is based on a graph-search algorithm. First, the area of interest, containing the next bone pixel, is defined. Knowing that the next bone pixel is to be found above and close to the current bone pixel root node (i.e., the first starting pixel at the far bottom of the image), the boundaries of the area of interest are set to 5 pixels above the current pixel and to 5 pixels to the left and the right hand side of the current pixel (as demonstrated in Figure 9a). The current pixel is marked as a red square, and all other pixels in the area of interest are marked as black squares. Next, the Euclidian distance is calculated from the current pixel to all other pixels in the area of interest whose intensity is larger than 0:

(6) $$D\left(P_{1},P_{2}\right)=\sqrt{{\left(P_{1x}-P_{2x}\right)}^{2}+{\left(P_{1y}-P_{2y}\right)}^{2}},$$

where $P_{1}$ and $P_{2}$ are pixels.

After calculating all the distances, the pixel closest to the root pixel is returned. In the cases where there are multiple pixels having the same distance value (as shown in Figure 9b), all of them are returned as a result and further processed by adding them to the graph.

#### 2.4.3. Building a Bone Graph

The procedure for building a graph that represents the bone edges starts from the root pixel and searches for the next pixels (according to the previously-explained algorithm), which are added to the graph as children of the root pixel (children of the root node of the graph). For every child node, the process is repeated (i.e., the next pixels are detected and added as nodes in the graph). The procedure is repeated until the algorithm finds a node that does not have any children, and all nodes are expanded. The last node is the one having the smallest horizontal axis coordinate, while the first node is the one having the largest horizontal axis coordinate.

**Figure 10:**
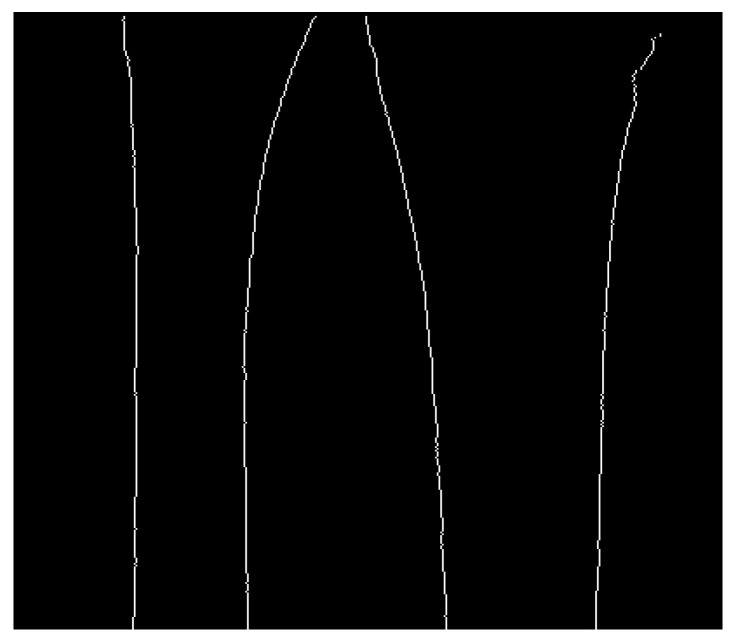
Bone contours filtered with the proposed algorithm.

Finding the proper path from the first node to the last node is performed by calculating all of the paths and choosing only the ones whose length is more than the preset percentage of the image height. Namely, this preset percentage value is the hyperparameter dependent on the patient age (based on the experiments on the training set, it was set to 60%). The resulting image was shown in Figure 10. Next, the last part of image preprocessing is to eliminate false bone contours.

#### 2.4.4. False Bone-Pixel Elimination

**Figure 11:**
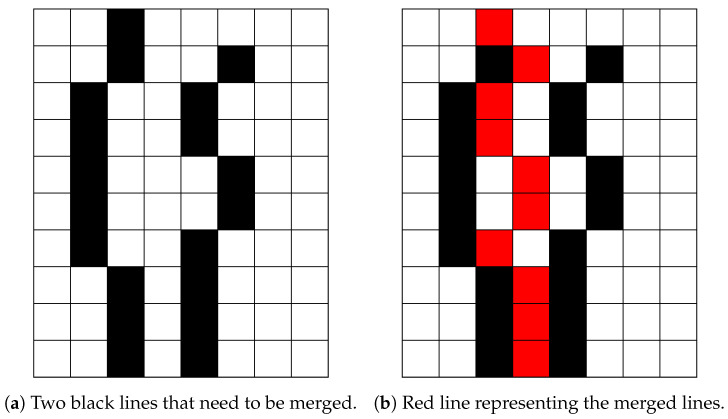
Merging lines.

The last problem in the proposed bone X-ray pre-processing pipeline is caused by blurry bones, which results in multiple lines for every bone contour. To solve this problem, a merging method, which combines multiple bone contours into one, has been proposed. It is important to notice that blurry bones, in general, cause several contours which are close to each other. For this purpose, we propose searching for additional contours in the vicinity of the initial one (i.e., inside a window of 15 pixels), which are then merged into one (on the row basis). Namely, for each row bone, pixels neighbouring the considered bone contour pixel (inside the widow width) are merged, and their mean pixel coordinate is set to represent the true pixel position. The described process is illustrated in Figure 11, where two black lines are merged into the red line.

### 2.5. Fracture Detection

The final, and most important, step of the proposed method is the fracture detection procedure (especially for small, demanding fractures). The algorithm calculates the error between the ideal bone line, and the bone line extracted from the captured X-ray image using the method described above. The ideal bone line can be considered as a smooth high-polynomial curve and, hence, polynomial regression was used to obtain the ideal healthy (i.e., fracture-free) bone contour. For polynomial regression to work, the curvature at the top of the bone must be removed (since it produces too much noise). To remove this part of the curvature, an algorithm is proposed for seeking the highest peak in the curvature and cropping the bone line. The curvature is measured by a second degree polynomial in the top 20% pixels of the bone line. Once the second degree polynomial is fitted to the top 20% pixels, the peak of the polynomial is set as the cropping point.

**Figure 12:**
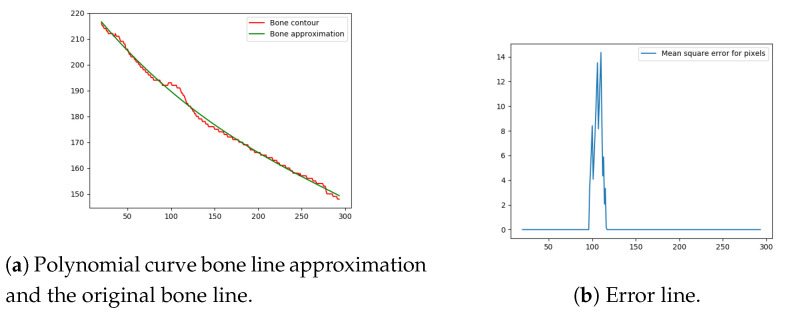
Bone line 1.

**Figure 13:**
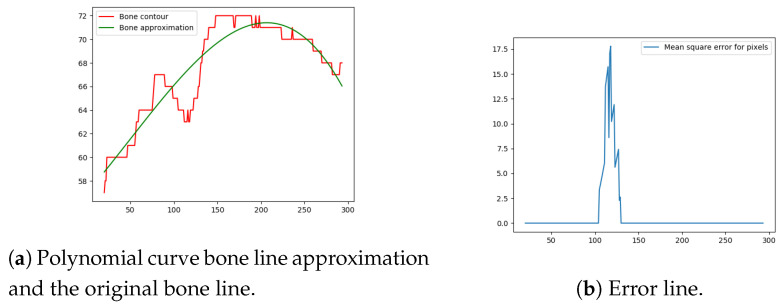
Bone line 2.

**Figure 14:**
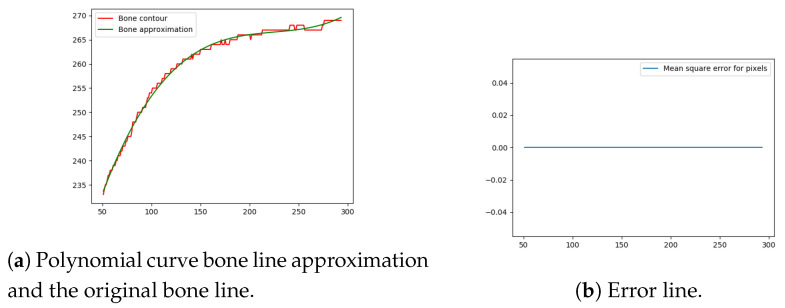
Bone line 3.

**Figure 15:**
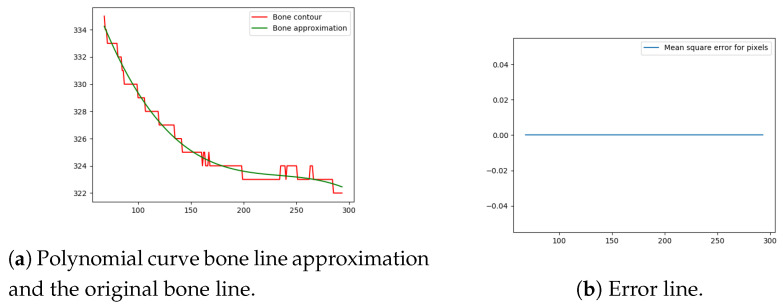
Bone line 4.

Next, the difference between the previously-estimated healthy bone line and the bone line extracted from the X-ray image is calculated, in terms of the mean squared error. The corresponding pixels are pixels having the same horizontal axis coordinates (as shown in Figure 12, Figure 13, Figure 14 and Figure 15), representing four bone lines of the two bones. Namely, the red line in the figure represents bone pixel intensities, while the green line represents the contour approximation of the ideal healthy bone obtained by polynomial regression (a 3rd order polynomial, due to the shape of the bone).

Next, the fracture in the bone was detected by tracking the mean squared error between the polynomial approximation and the original bone contour. Based on the analysis performed on the training data set, and taking into the account the blurred bone imperfections and inaccuracies in contour pixel position estimation caused by bone merging, a tolerance of 3 pixels was implemented.

**Figure 16:**
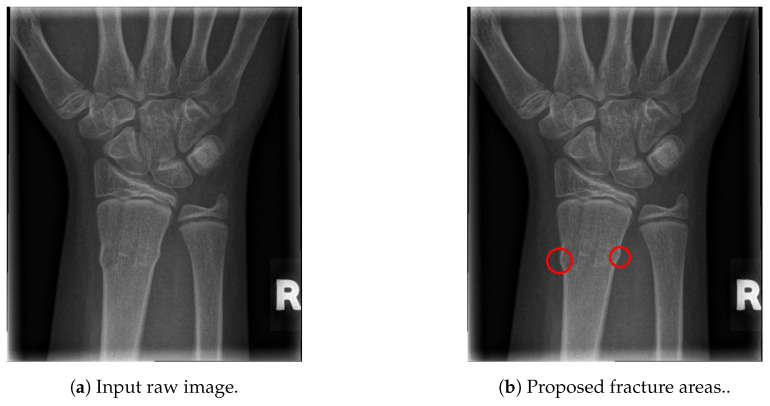
Fracture area detection.

The final fracture detection and localisation results are shown in Figure 12, Figure 13, Figure 14 and Figure 15 (denoted by blue lines). Figure 12 and Figure 13 show two peaks which represent potential fractures. The critical fracture area is marked in the X-ray image by a red circle (at the centre of which is the coordinate of the highest peak, with radius being defined by the width of the area), as shown in Figure 16.

As can be seen, the proposed method was shown to be efficient, not only in detecting the fractures in the images (flagging the images as either containing fractures or fracture-free), but also precisely localising the position and the size of the region with the fracture. This can be used to assist medical staff to consider only the images with fractures and to direct their attention to specific regions of interests containing demanding fractures.

In addition, one of the advantages of the proposed method is its possible application in labelling large data sets of medical images (with accurate specification of the fracture positions) to be used for training classification models based on neural networks and deep learning (supervised learning). This could enable an even more refined classification of fractures in the regions already detected by the method proposed in this paper. However, neural network-based classification is out of scope of this paper and is planned to be investigated in our future work.

In particular, the proposed hybrid method may be considered as a useful tool and an important step towards the replacement of tedious X-ray image labelling, done manually by medical experts, by the automated computerised method proposed in this paper.

## 3. Results

The proposed method for the detection of smaller, demanding fractures of the radius and ulna bones from X-ray images, based on the local entropy, was applied to a data-set consisting of 960 coronal plane images of child radius and ulna bone fractures provided by the Medical University of Graz, Department of Radiology, Division of Paediatric Radiology. Note that all images of open fractures or shattered bones (easy to detect even for a non-medical observer) were removed from the initial data set, as those kind of injuries were out of the scope of the proposed algorithm. In particular, out of the 860 images that remained in the tested data-set (100 images were used as the training set), 218 images contained fractures, and 642 were fracture-free. A F1 score, with the following notation, was used as a classification performance metric:

- **True Positive (TP)**: The case when the image contains a fracture and the algorithm marked it as fractured, and at least one of the present fractures is correctly marked.
- **False Negative (FN)**: The case when the image contains a fracture, but the algorithm failed to mark the image as fractured.
- **True Negative (TN)**: The case when the image does not contain a fracture and the algorithm did not mark it as fractured.
- **False Positive (FP)**: The case when the image does not contain a fracture, but the algorithm marked it as fractured and marked a fracture that is not present.

The proposed method was first fine-tuned on the training set consisting of 100 images, 13 of which contained fractures and 87 which were without fractures. The classification performances of the proposed method on the training set and on the test set are given in Table 2.

Out of the 218 images containing fractures in the test set, 169 were detected or marked correctly as fractured images, and 49 were mis-classified (fracture was not detected or marked). Next, out of 642 images that did not contain fractures, 615 images were correctly classified, and only 27 images were mis-classified (meaning that the algorithm falsely detected a fracture that was not present).

The main reason for fractures not being detected is that there are many types of fractures, and some of them do not produce a detectable difference in the bone contour and so do not trigger the proposed detection algorithm. The proposed algorithm can be tuned with several hyperparamters, and their fine-tuning can result in enhanced classification performances to correctly classify even the smallest deviations in bone contour (on the other hand, this leads to an increase in the number of false positives). In addition, the main reason for false positive detections is blurriness of the bones, which results in irregular bone contours after the image segmentation step.

Sensitivity and specificity measure the algorithm’s performance inside each class. Sensitivity measures how well the algorithm performed on images containing fractures, while specificity measures how well the algorithm performed on images not containing fractures. As shown in Table 2, the algorithm performed better on images not containing fractures (95.79% against 77.52%). The false positive rate, false negative rate, and false discovery rate measure the number of mistakes the algorithm makes, so lower numbers (ideally 0) of these rates are desirable. In terms of these measures, the proposed algorithm also performed well, with 4.21% false positive, 22.48% false discovery, and 13.78% false negative rates.

The most important measures—precision, accuracy, and F1 Score–showed the true ability of the algorithm to detect fractures. As can be seen in Table 2, both precision and accuracy were reasonably high (86.22% and 91.16%), which can be interpreted as follows: The proposed algorithm was correct in 86.22% of the cases in marking images as fractured (precision); while, of all the available images in the data-set, the algorithm correctly classified 91.16% of them (accuracy). Combining the precision and recall/accuracy, the F1-score (81.64%) shows that the proposed algorithm is capable of making correct suggestions to the radiologists, in terms of marking fractures that can be easily overseen.

In addition, just for comparison, instead of using the proposed entropy-based segmentation method, fracture detection was performed using Canny filter segmentation, which resulted in an accuracy of 61.86%, precision of 46.75%, and a F1 score of 49.07%. As can be seen, even though the Canny filter performed competitively in terms of segmentation (unlike the proposed method), it was rather inefficient in fracture detection.

The proposed method is also of great help in labelling fractured images by marking the exact fracture location (unlike some of the existing approaches, which only label medical images as fracture-free or containing fractures). Once the images are labelled with the exact fracture location, a neural network can be trained for even more refined fracture classification. Namely, employing neural networks is the next step of our future work towards a comprehensive fracture detection method from X-ray images.

**Figure 17:**
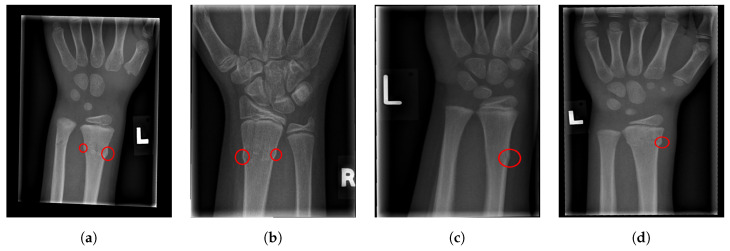
Examples from the true positive (TP) performance metric class.

**Figure 18:**
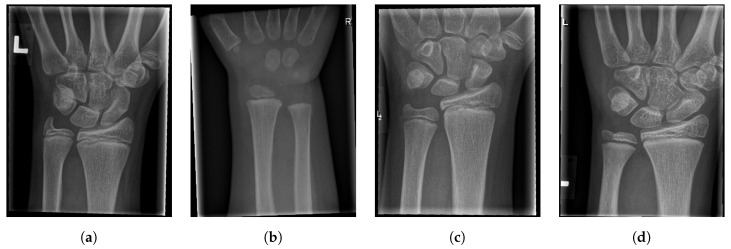
Examples from the true negative (TN) performance metric class.

**Figure 19:**
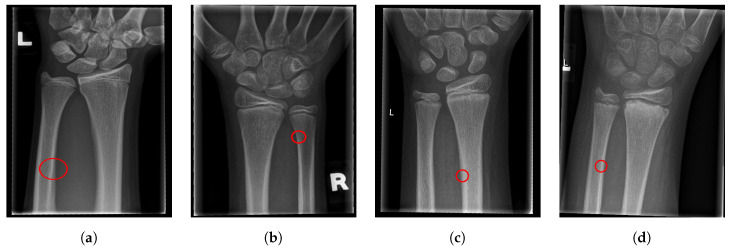
Examples from the false positive (FP) performance metric class.

**Figure 20:**
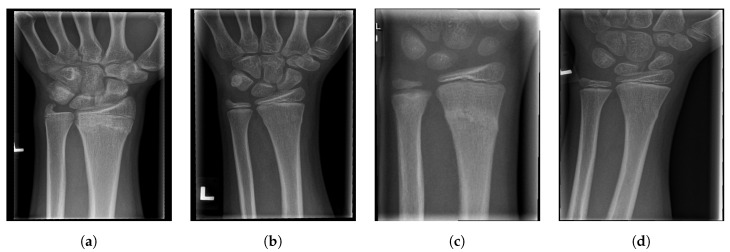
Examples from the false negative (FN) performance metric class.

For illustration, four images from each class (TP, TN, FP, and FN) are given in Figure 17, Figure 18, Figure 19 and Figure 20.

## 4. Conclusions

The proposed algorithm, based on the local entropy, successfully classified and precisely marked the positions of small, demanding bone fractures in child ulna and radius bones with an accuracy of 91.16%. The hybrid multi-stage method consists of the following steps: First, the rotation-invariant property of the method is achieved by a PCA-based procedure. Next, bone segmentation and tissue removal is achieved, by utilising a novel technique based on the local entropy (shown to outperform other filters, such as Laplacian, Sobel, and Canny filters). Once the tissue is removed, the region of interest, containing ulna and radius bones, is extracted based on row peak-values. Next, a graph theory-based algorithm is introduced for determining bone contours. Finally, an algorithm for detection of small, demanding fractures is proposed, by comparing the ideal healthy bone estimation and extracted real bone contours. The proposed hybrid approach is a novel pipeline, which efficiently both detects and localises fractures in X-ray images of long bones. The proposed technique was tested on 860 X-ray images (218 images containing fractures and 642 fracture-free images). The achieved results (in terms of precision, sensitivity, specificity, and F1 score) demonstrate the efficiency of the proposed approach in detecting small, demanding fractures in the ulna and radius bones. However, improved fracture detection and localisation is expected to be achieved through the utilisation of deep neural networks, which will be investigated in our future work. However, the proposed method can replace tedious manual X-ray image labelling with the automated computerised solution proposed in this paper. Once labelled, this data is planned to be used for training classification models based on neural networks and (supervised) deep learning.

## Figures and Tables

**Table 1:** Segmentation results for different algorithms.

Image	Image Entropy H(X)	Segmentation Entropy E(S)	Global Entropy N
Raw image	0.82695	0.78989354	1.6168
Canny filter	0.41546	0.59832	1.0138
Laplacian filter	4.92800	0.67138	5.5994
Vertical Sobel filter	1.01651	0.85196	1.8685
Horisontal Sobel filter	1.03558	0.79135	1.8269
Proposed filter	0.236274	0.61663	0.85290

**Table 2:** Results of the algorithm performance on the training and test sets.

Measure	Train Set Values	Test Set Values	Derivations
True Positive	12	169	TP
True Negative	84	615	TN
False Negative	1	49	FN
False Positive	3	27	FP
Sensitivity	0.9231	0.7752	$TPR=\frac{TP}{TP+FN}$
Specificy	0.9655	0.9579	$SPC=\frac{TN}{FP+TN}$
Precision	0.8000	0.8622	$PPV=\frac{TP}{TP+FP}$
Negative Predictive Value	0.9882	0.9262	$NPV=\frac{TN}{TN+FN}$
False Positive Rate	0.0345	0.0421	$FPR=\frac{FP}{FP+TN}$
False Discovery Rate	0.2000	0.1378	$FDR=\frac{FP}{FP+TN}$
False Negative Rate	0.00769	0.2248	$FNR=\frac{FN}{FN+TP}$
Accuracy	0.9600	0.9116	$ACC=\frac{TP+TN}{P+N}$
F1 Score	0.8571	0.8164	$F1=\frac{2TP}{2TP+FP+FN}$
